# Optimization of catalytic properties of *Mucor racemosus* lipase through immobilization in a biocompatible alginate gelatin hydrogel matrix for free fatty acid production: a sustainable robust biocatalyst for ultrasound-assisted olive oil hydrolysis

**DOI:** 10.1007/s13205-022-03319-8

**Published:** 2022-09-19

**Authors:** Heidi Mohamed Abdel-Mageed, Dina Nada, Rasha Ali Radwan, Saleh Ahmed Mohamed, Nesrine Abdelrehim E. L. Gohary

**Affiliations:** 1 Molecular Biology Department, National Research Centre (NRC), El Behoth St Dokki, Cairo, Egypt; 2 Pharmacology and Biochemistry Department, Faculty of Pharmacy, The British University in Egypt (BUE), Cairo, Egypt; 3 Center for Drug Research and Development (CDRD), The British University in Egypt (BUE), Cairo, Egypt; 4 Pharmaceutical Chemistry Department, Faculty of Pharmacy and Biotechnology, German University in Cairo (GUC), Cairo, Egypt

**Keywords:** Alginate gelatin hydrogel, Biocatalyst, Oleic acid, Ultrasound, Enzyme immobilization, Olive oil hydrolysis, Biocompatible, Green technology

## Abstract

Immobilization is a key technology that improves the operational stability of enzymes. In this study, alginate-gelatin (Alg-Gel) hydrogel matrix was synthesized and used as immobilization support for *Mucor racemosus* lipase (Lip). Enzyme catalyzed ultrasound-assisted hydrolysis of olive oil was also investigated. Alg-Gel matrix exhibited high entrapment efficiency (94.5%) with a degradation rate of 42% after 30 days. The hydrolysis of olive oil using Alg-Gel-Lip increased significantly (*P* < 0.05) as compared to free Lip. Optimum pH and temperature were determined as pH 5.0 and 40 °C, respectively. The Vmax values for free and immobilized Lip were determined to be 5.5 mM and 5.8 mM oleic acid/min/ml, respectively, and the Km values were 2.2 and 2.58 mM/ml respectively. Thermal stability was highly improved for Alg-Gel-Lip (t_1/2_ 650 min and Ed 87.96 kJ/mol) over free Lip (t_1/2_ 150 min and Ed 23.36 kJ/mol). The enzymatic activity of Alg-Gel-Lip was preserved at 96% after four consecutive cycles and 90% of the initial activity after storage for 60 days at 4 °C. Alg-Gel-Lip catalyzed olive oil hydrolysis using ultrasound showed a significant (*P* < 0.05) increase in hydrolysis rate compared to free Lip (from 0.0 to 58.2%, within the first 2 h). In contrast to traditional methodology, using ultrasonic improved temperature-dependent enzymatic catalyzed reactions and delivered greater reaction yields. Results suggest that Alg-Gel-Lip biocatalyst has great industrial application potential, particularly for free fatty acid production. In addition, the combined use of enzyme and ultrasound has the potential of eco-friendly technology.

## Introduction

Numerous industries depend on the hydrolysis of oils and fats to produce fatty acids which are essential components in the manufacturing of many products including coatings, surfactants, adhesives, flavors, cosmetics, food, detergents, and others (Alves et al. 2014). The Colgate-Emery process commonly used for the hydrolysis of oils and fats is a steam treatment technology with high temperature (250 OC) and high pressure (70 bar) (Barnebey and Brown 1948). Yet, due to severe reaction conditions, this technology causes undesired reactions such as oxidation, dehydration, and inter-esterification of triglycerides (Alves et al. 2014; Ferreira et al. 2019). The steam treatment is also incompatible with heat-sensitive oils and oils containing polyunsaturated fatty acids (Chen et al. 2014). The use of enzymes for oil hydrolysis, therefore, presents a more cost-effective and easily-operated method that has high fatty acid selectivity and only requires mild conditions, such as atmospheric pressure (1013.25 mbar) and low temperatures between 30 and 45 degrees Celsius. Hence, all of the disadvantages associated with the traditional hydrolysis techniques are evaded (Aziz et al. 2015). In addition, the separation of fatty acids and glycerol is rather a relatively simple process after the enzymatic hydrolysis. Thus, biocatalyst enzyme systems are increasingly valued for their high-efficiency and eco-friendly advantages. On the other hand, Olive oil is a common commercial edible oil that would serve as an economical source for free fatty acids production. Olive oil is also characterized by its high content (65 to 85%) of oleic acid, which is the major component among monounsaturated fatty acids (MUFA), as well as other fatty acids such as linoleic, palmitic and stearic acids (Ben Ayed et al. 2018). Likewise, Lipases (triacylglycerol hydrolases E.C. 3.1.1.3) are exceptionally valuable hydrolytic enzymes with a wide range of bio-catalytic, biomedical, and industrial applications. They have high catalytic activity and specificities that are free of unwanted by-products. These characteristics have greatly increased their application as biocatalysts in several industries (Abdel-Mageed et al. 2021a). Lipases can be obtained from a variety of sources including animals, vegetables, and microbes. However, in comparison to animal and plant lipases, microbial lipases are subject of interest due to their lower production costs, long-term stability under processing conditions, and availability (Abdel-Mageed et al. 2021b). In this study, the Lipase used was isolated from Mucor sp., it was purified and characterized as yielding a high specific activity of 15,217 units/mg protein, optimum pH at 5.0 and temperature optimum of 40 °C. In addition, the purified lipase was shown to be stable up to 70 °C. These characteristics are previously reported by the research team (Abdel-Mageed et al. 2020; Mohamed et al. 2011).

Biocatalysts must be easily extracted from the reaction mixture, reusable without loss of activity, and stable to be economically beneficial for industrial use (Chandra et al. 2020). Immobilization of enzymes has emerged as a feasible method for achieving this aim using various approaches. Different adsorption methodologies or covalent binding techniques employing linking chemicals, encapsulation and/or entrapment in gel-like materials, cross-linking of enzymes, or a combination of these methods can be used to attach enzymes to supporting matrices by physical or chemical means (Abdel-Mageed et al. 2019a, 2019b; Wahab et al. 2020). The method for immobilizing enzymes can, however, have a significant impact on the overall feasibility of industrial applications and must therefore meet certain criteria. Resistance to microbial contamination is an important factor that must be considered (Wahab et al. 2020). In addition, and in order to maximize the economic impact of the total process, the method employed has to be of low-cost and ecologically benign (Abdel-Mageed et al.^2020^; Abdel-Mageed et al. 2021b). It should also manifest the greatest potentials in relation to enzyme stability and activity. The use of a hydrogel matrix to immobilize enzymes has risen in popularity in this context as it demonstrates biocompatibility and biodegradability. It is also an environmentally benign support matrix with minimum toxicity. Alginate, an anionic polysaccharide isolated from the cell walls of seaweeds and brown algae, is a linear copolymer containing the residues -l-guluronic acid (G) and -d-mannuronic acid (M). Because of its good biocompatibility, simplicity of handling and processing, as well as its excellent gel porosity, alginate has been frequently used for enzyme immobilization (Zhao et al. 2015), where the Ca^+2^ ions physically cross-linked alginates, generate an egg-box-like shape (Labus et al. 2020). Yet, alginate networks are flexible and swell quickly due to the action of inner osmotic pressure which causes partial leakage of the entrapped enzymes from the polymeric supports. The mechanical characteristics and robustness of alginate-based supports are thus improved by blending alginate with other matrices (Labus et al. 2020). Various alginate hybrid support systems are studied in this context, including chemically reduced graphene oxide-alginate microbeads for entrapping glucose oxidase (Zhao et al. 2015), polyvinylpyrrolidone-alginate microspheres (Hwang et al. 2011), boehmite-alginate beads (Ai et al. 2013), chitosan-alginate beads (Pereira et al. 2019), gelatin-alginate fibers (Naganagouda and Mulimani 2006) and alginate-gelatin spheres (Mörschbächer et al. 2016)

Gelatin is made up of proteins and peptides that are formed when collagen is partially hydrolyzed and broken down into smaller fragments. One downside of gelatin is its reversible gelation, which renders it effectively immobilized only at temperatures between 30 and 35 °C. Various strategies including the use of cross-linking agents have been investigated to achieve an irreversible gelation process (Abdel-Mageed et al. 2022b; Chen et al. 2020). In this study, glutaraldehyde was chosen as the cross-linker agent.

Furthermore, in coping with the paradigm shift toward green chemistry and sustainable processes, ultrasound has received a special attention in the optimization of enzyme-catalyzed reactions. Several studies have proposed that the energy released by ultrasound during the phenomenon known as cavitation can be utilized to boost mass transfer (substrate/enzyme), hence enhancing the rate of product production and improving enzyme catalytic activity due to boosted substrate accessibility to the enzyme active site (Souza et al. 2020). In addition, when compared to other techniques, Ultrasound can be viewed as Green technology resulting in a high economic value as a process because of its supreme efficiency with just one-third to half of the energy used by mechanical agitation, low equipment requirement, and significant processing time savings (Lerin et al. 2014). In industry, the combined use of enzyme-catalyzed processes and ultrasound might as well be potentially an eco-friendly technology. Thus, in terms of environmental protection, such implementation tends to be a promising clean technology.

This study aims mainly to physically immobilize the lipase enzyme produced from *Mucor racemosus* using the alginate-gelatin hydrogel matrix. The immobilization yield, pH and temperature optima, kinetic characteristics, reusability, and storage stability are investigated. In addition, the catalytic methodology for olive oil-assisted ultrasonic and various process parameters are examined. Although, various studies on olive oil hydrolysis have been published, to the authors’ best knowledge, there is limited data on ultrasound-assisted enzyme-catalyzed olive oil hydrolysis, particularly when Mucor lipase is used. Moreover, the combination of the immobilized enzyme with ultrasound is considered Green technology which increases profoundly the overall significance of this study.

## Materials and methods

### Materials

Lipase (glycerol ester hydrolase EC 3.1.1.3) is purified and characterized in the lab from *Mucor racemosus* Nrrl 3640 (0.23 mg protein; 15,217 units/mg protein) **(**Mohamed et al. 2011**)**. P-nitrophenyl palmitate (p-NPP), gelatin (Type B, porcine), glutaraldehyde, alginate (Sodium alginate), calcium chloride of analytical grade, and all other chemicals of reagent grade are purchased from Merck (GmbH, Darmstadt, Germany). Gum Arabic, olive oil is purchased from Sigma Aldrich Chemical Co., St. Louis, MO, USA.Ultrasound equipment used in all experiments (Elmasonic P ultrasonic, P70H, Thomas Scientific, Germany) was of a capacity volume of 6.9 L and dimensions of 505 x 137 x 100 mm (length x width x height). The ultrasound frequency was 37 kHz, with a total power of 300 W.

### Immobilization procedure of lip in the hydrogel matrix

Lip was immobilized by entrapping in the Alg-Gel hydrogel matrix with modifications to the previously reported methodology (Abdel-Mageed et al. 2022b). After alginate and gelatin (2%W/V) were dissolved in potassium phosphate buffer (pH:7.0, 50 mM) at 45 °C, a homogenous mixture solution was prepared. The solution is cooled down and 25% glutaraldehyde solution was used for covalent crosslinking of gelatin molecules followed by mechanical stirring for 6 h at 1000 rpm. Afterward, 3% (w/v) calcium solution was added for ionic crosslinking of alginate molecules and the solution was agitated for 30 minutes at low stirring so as to produce the hydrogel matrix (Alg-Gel). For the immobilization of Lip, 2 mL Lip (15000 U/mL, in Tris–HCl buffer; pH:7.0, 50 mM) was added to the solution and stirred for 10 min (Alg-Gel -Lip). The formed gels were washed with distilled water and stored at 4 °C. The obtained gel matrix was divided into small disks of Lip content of 3000 U/g for further investigations.

### Degradation test

The prepared hydrogel matrix is subjected to degradation tests at pH 7.4 and 37 °C (Tan et al. 2009; Pulat and Akalin 2013). Dried hydrogel disks were allowed to swell in phosphate buffer saline. After 48 hours, swollen gels were separated from the swelling medium, carefully dried with filter paper and then weighed. The maximum swollen state of hydrogels is assumed to be the reported mass (Mo). The samples are all reintroduced into the same bath and weighed at regular time intervals until the hydrogels are completely degraded. The degradations (%) are determined using this equation (1):

1 $$\mathrm{Degradation \%}=\frac{\mathrm{Mo}-\mathrm{Mt}}{\mathrm{Mo}}\times 100$$

In the equation, Mo denotes the weight of the hydrogel at its fully swollen state and Mt denotes the weight of the hydrogel at time t. All measurements are in triplicate.

#### Lip hydrolytic activity assay

Hydrolytic activities of free Lip and Alg-Gel-Lip are assayed using the olive oil emulsion hydrolysis method with modifications in accordance with previous publications (Mohamed et al. 2011). The substrate emulsion is prepared using 25 ml of 50% (w/v) gum acacia/water and 15 ml of 10 mM Tris–HCl buffer, pH 8.0 with 10 ml of olive oil (as the substrate) with the final pH adjusted to 5.5. Two procedures were employed to investigate olive oil hydrolysis catalyzed by the addition of either free Lip or immobilized Alg-Gel-Lip, in a solvent-free system. 20 mL of emulsified olive oil was added to a 150 cm^3^ conical flask to initiate the hydrolytic processes. To homogenize the reaction mixture, mechanical stirring (200 rpm) and/or ultrasound at the specified power (37 kHz/300 W) were used. Each reaction mixture was incubated for 60 minutes at 40°C under atmospheric pressure. The hydrolysis reactions are then stopped by adding 10 mL of acetone and ethanol solution (1:1). The liberated free fatty acids (FFA) were thereafter titrated in the presence of phenolphthalein as an indicator in a 20 mM sodium hydroxide solution. Blanks experiments were carried out by titrating 1 mL of substrate emulsion without Lip sample. Under standard assay conditions, one international unit (U) of Lip activity was defined as the amount of enzyme required to liberate 1 µmol of FFA per minute.

#### Determination of protein concentration

Protein concentration analyses were performed using bovine serum albumin (BSA) as standardized and described by (Bradford (1976)).

### Immobilization yield

For the determination of the immobilization yield, 3 mm^3^ homogenized cubes of Alg-Gel-Lip matrix were incubated in the substrate mixture to determine the immobilization yield, which was considered as the ratio of enzymatic activity assayed in the hydrogel matrix to the total units of the free enzyme under standard assay conditions. The immobilization yield percentage is calculated as per equation (2):

2 $$\mathrm{Immobilization yield}\left(\mathrm{\%}\right)=\frac{\mathrm{Total activity of immobilized Lip }}{\mathrm{Total initial free enzyme activity }}\times 100$$

### Enzymatic hydrolysis of olive oil

Enzymatic hydrolysis of olive oil was performed with modifications according to the method reported by Ferreira et al. (2019). The reaction mixture was prepared in 125 mL Erlenmeyer flasks containing 45 ml of reaction medium made up of 15 g of emulsified olive oil, 30 g 0.1 M Tris-HCl buffer solution, pH 7.0. The final olive oil concentration (6 mM) in the enzymatic reaction medium was calculated according to its content in oleic acid.

To initiate the enzymatic reaction, either 1 mL of free Lip solution (3000 U/mL) prepared in 0.1 M Tris-HCl buffer solution at pH 7.0, or 1 g of Alg-Gel-Lip (equivalent to 3000 U) was added directly to the reaction mixture. The Alg-Gel-Lip gel matrix was homogenized for 10 seconds with the Ultrasound Sonicator before being added to the reaction mixture to achieve a homogenize mixture. The biocatalysts assay mixtures were then incubated for 1 hour at 40 °C, either in an ultrasound bath or subjected to continuous mechanical stirring (200 rpm) for 1 hr. A control experiment was thereafter conducted, which includes all reaction mixture components aside from the enzyme preparation.

Afterwards, samples (1 g of the reaction mixture) were taken from the reaction mixture at regular intervals and placed in a 100 ml conical flask containing a 10 mL solution of ethanol and acetone (1:1) to stop enzymatic reaction. The flask was shaken in hot water to ensure the complete dissolution of the sample. Following dissolution, the samples are titrated with a standardized 20 mM NaOH solution while using phenolphthalein as an indicator, whereas the transition to pink indicated the endpoint of the titration. Every reaction was carried out in triplicate. The degree of hydrolysis was calculated according to Rooney and Weatherley (2001) where the percentage weight of FFAs in the sample is divided by the initial weight of the olive oil sample, as shown in equation (3):

3 $$\mathrm{Hydrolysis }(\mathrm{\%})=\frac{{\mathrm{V}}_{\mathrm{NaOH}}\times 10-3\times {\mathrm{M}}_{\mathrm{NaOH}}\times \mathrm{MWt}}{\mathrm{Wt}\times \mathrm{f}}\times 100$$

In the equation, V denotes the volume of NaOH solution required for titration; M denotes the concentration of NaOH (20 mM), MWt denotes the molecular weight of linoleic acid (280.45 g/mol), Wt denotes the initial weight of the olive oil sample and f denotes the oil fraction at the start of the reaction.

#### Effect of enzyme loading

At a constant reaction time of 3 hours, the effect of enzyme concentration, 0.0 to 5.0 mg free Lip or Alg-Gel-Lip solid, on the hydrolysis of olive oil was investigated under standard assay conditions.

### Comparative analysis of mechanical stirring vs ultrasound-assisted enzymatic hydrolysis of olive oil using immobilized lip

#### Effect of reaction time

The effect of reaction time on olive oil hydrolysis under either mechanical stirring or ultrasound using time intervals of 0 to 6 h was determined using Alg-Gel-Lip as the biocatalysts. Residual lip activity was further investigated under standard assay conditions.

#### Effect of temperature

The effect of varying temperatures on olive oil hydrolysis under either mechanical stirring or ultrasound using different temperatures –ranging from 40 to 60 °C for 3h—was determined using Alg-Gel-Lip as the biocatalysts. Residual Lip activity was further investigated under standard assay conditions.

### Characterization of free lip and immobilized Lip

#### Effect of PH

The effect of pH on free Lip and Alg-Gel-Lip was investigated using different buffer solutions: (0.05 M Glycine–HCl (3.0–4.0 pH), sodium acetate (4.0–5.0 pH), sodium succinate (6.0–6.5 pH), potassium phosphate (6.5–8.0 pH), and Tris–HCl (8.0–9.0 pH) under standard assay conditions. Optimum pH is considered as a 100% catalytic activity whereas other activities were expressed as a percentage of this optimum 100% activity.

#### Effect of temperature

The temperature profile for free Lip and Alg-Gel-Lip was investigated by examining the catalytic activity at temperatures ranging from 20 to 90 °C with an interval of 10 °C. The assay for residual enzyme activity was concurrently performed under standard assay conditions.

#### Thermal stability

Using the thermal inactivation method at various temperatures, the effect of temperature on enzyme stability for free Lip and Alg-Gel-Lip was determined. Soluble lipase (1 mg of crude lipase extract, corresponding to 0.23 mg of protein) is incubated at 40, 50, and 60 °C for 180 min in the absence of a substrate. Samples were then collected at predetermined intervals and immediately cooled in an ice bath for 5 minutes. On the other hand, the residual enzyme activity was determined using the olive oil emulsion hydrolysis assay, as previously described.

The first order thermal inactivation rate constants K_d_ was calculated according to the following equation (4):

4 $$LnA=LnAo-{K}_{d}t$$

In the equation, Ao is the initial enzyme activity and A is the residual activity after time t. By plotting a graph of −ln A/A_0_ on Y-axis against time (t) on the X-axis, the slope of the graph gives K_d_ of the enzyme.

Sadana and Henley's (1987) decay model is used to calculate thermal inactivation constants (k_d_) and half-lives (t_1/2_). The activation energy for thermal denaturation of free and immobilized Lip is calculated using the Arrhenius equation employing a plot of ln k_d_ as a function of 1/T and the data were then fitted according to the following equation (5):

5 $$\mathrm{lnKd}=\mathrm{lnA}-\frac{{\mathrm{E}}_{d}}{\mathrm{R}}\times \frac{1}{\mathrm{T}}$$

In the equation, *A* is the Arrhenius frequency of collision factor (*E*_d_) denotes the activation energy for thermal denaturation (kJ/mol), (*R)* stands for universal gas constant (8.314 × 10^−3^ kJ/mol K), and *T* denotes the absolute temperature (K).

The t_1/2_ was calculated using equation (6):

6 $${\mathrm{t}}_{1/2}=\frac{0.693}{{K}_{d}}$$

#### Determination of the kinetic parameters

The kinetic constants K_m_ V_max_ catalytic efficiency (V_max_/K_m_) of free and immobilized Lip were determined using Lineweaver–Burk plots using the graph pad prism program (version 5.0) and by measuring the enzyme activity at different concentrations of substrate (olive oil as a substrate (5–80 mg/L)) and thus determining the FFA produced. On the other hand, the apparent K_m_ and V_max_ values for the free and immobilized Lip were determined using Lineweaver–Burk plots by employing the initial rate of the lipase reaction given according to the following equation (7):

7 $$\mathrm{V}=\frac{{\mathrm{V}}_{\mathrm{max}} [\mathrm{S}]}{{\mathrm{K}}_{\mathrm{m}}+\mathrm{S }}$$

In the equation, [S] is the substrate concentration, *V* and *V*_max_ represent the initial and maximum rate of reaction respectively, and *K*_m_ is the Michaelis–Menten constant. The K_m_ is calculated using the Lineweaver–Burk plot which presents a linear relationship between 1/V and 1/S respectively.

The apparent parameter k_cat_ was calculated using the following equation, where [Et] is the lipase concentration (0.00184 μmol/mL) (Abdel-Mageed et al. 2019b) (8):

8 $${\mathrm{K}}_{cat}=\frac{{V}_{max}}{[\mathrm{Et}]}$$

#### Re-use of immobilized Lip

The reusability of Alg-Gel-Lip was evaluated during consecutive hydrolysis reactions that were carried out under standard assay conditions. After the initial hydrolysis reaction, the Alg-Gel-Lip gel matrix disks were removed from the reaction medium by vacuum filtration and the biocatalyst was washed with distilled water three times. Afterward, consecutive hydrolysis cycles were initiated to evaluate the reusability of the immobilized Lip. This procedure was repeated for 6 consecutive cycles.

### Storage stability

For 60 days, free and immobilized Lip were stored at 4 °C to determine the enzyme’s storage activity. Samples were collected for activity assay at predetermined time intervals. Residual Lip activity was then investigated under standard assay conditions.

### Statistical analysis

All enzymatic activity assays were carried out in triplicate and the obtained data are reported as mean ± SD. Data analysis was carried out using one-way ANOVA. Statistical analysis was performed using SPSS software 19.0 version (SPSS Inc., Chicago, IL, USA). Differences were considered statistically significant at *p* < 0.05.

## Results and discussion

### Hydrogel matrix formation and immobilization yield

It is vital for industrial applications to create a stable biocatalyst with maximum mechanical and operational strength. In this study, therefore, mild processing conditions were employed for Lip immobilization in Alg-Gel hydrogel that was prepared using 2% W/V gelatin and sodium alginate. In addition, an Alg-Gel hydrogel was synthesized and cross-linked with glutaraldehyde and Ca^2+^ ionic crosslinking. Glutaraldehyde covalent crosslinking is typically introduced before Ca^2+^ ionic crosslinking so as to obtain a homogeneous internal microstructure (Chen et al. 2020).

Alginate hydrogels are synthesized in the presence of divalent cations such as Ca^2+^ in an aqueous solution, which serve as ionic crosslinking agents. It is proposed that Ca^2+^ interacts with the biopolymer's G block by substituting the sodium ions in the G-blocks therein, generating the well-known egg-box structure. The commonly used crosslinking agent to improve mechanical strength and long-term stability of gelatin-based hydrogels is glutaraldehyde. Furthermore, ionic interactions between sodium alginate and gelatin arise due to the presence of ionizable amino and carboxyl groups whereas hydrogen interactions between the amine and carboxyl group occur (Li et al. 2022). A potassium phosphate buffer was used in this study to slow and control the gelation rate, which is essential to generate consistent hydrogels with greater mechanical strength. The phosphate groups in the buffer are thought to compete with the –COOH group of Alginate during the reaction with calcium ions resulting in a regulated gelation process. In addition, both sodium alginate and gelatin act directly as enzyme stabilizers (Abdel-Mageed et al. 2022a). Furthermore, lower temperatures result in a decreased divalent cation reactivity rate, ensuing a highly ordered cross-linked gel matrix that increases mechanical characteristics as well (Abasalizadeh et al. 2020).

Physical immobilization thru entrapment is a gentle technique that has no effect on the enzyme conformational shape and so, retains its catalytic activity. High entrapment efficiency of Lip in Alg-Gel hydrogel was determined (94.5%). It is proposed that the mild immobilization process used during hydrogel matrix formation preserves and protects Lip structure in the biopolymer gel. The high entrapment efficiency of enzymes (≥ 80%), upon physical immobilization, has been described by several authors using different systems such as vesicles (Abdel-Mageed et al. 2012, 2018a); hydrogels (90%, Osuna et al. 2015), as well as chitosan beads (78%, Yagar and Balkan, 2017).

### Degradation behaviors of the hydrogel matrix

**Fig. 1:**
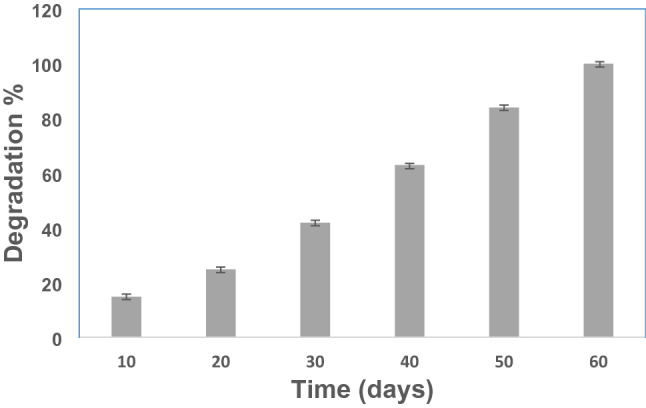
The variation of degradation % values for prepared cross-linked hydrogel matrix (Gel-Alg) (2% (w/v) measured at 37 °C, pH 7.4. Each point reflects the average of three experimental results ± SD

Alginate and gelatin are natural biodegradable polymers. Therefore, Alg-Gel hydrogel/lipase matrix was investigated in this study for industrial applications, where degradation profiles of the hydrogel matrix are considered a principal criterion. Figure 1 presents the degradation behaviors of Alg-Gel hydrogels at 37 °C and pH 7.4. The degradation rate exhibited a slow increase during the first 15 days with less than 20% degradation. The prepared hydrogel matrix showed good stability with a degradation rate of 42% after 30 days.

### Physicochemical characterization of free and immobilized Lip

#### Effect of enzyme concentration on olive oil hydrolysis

**Fig. 2:**
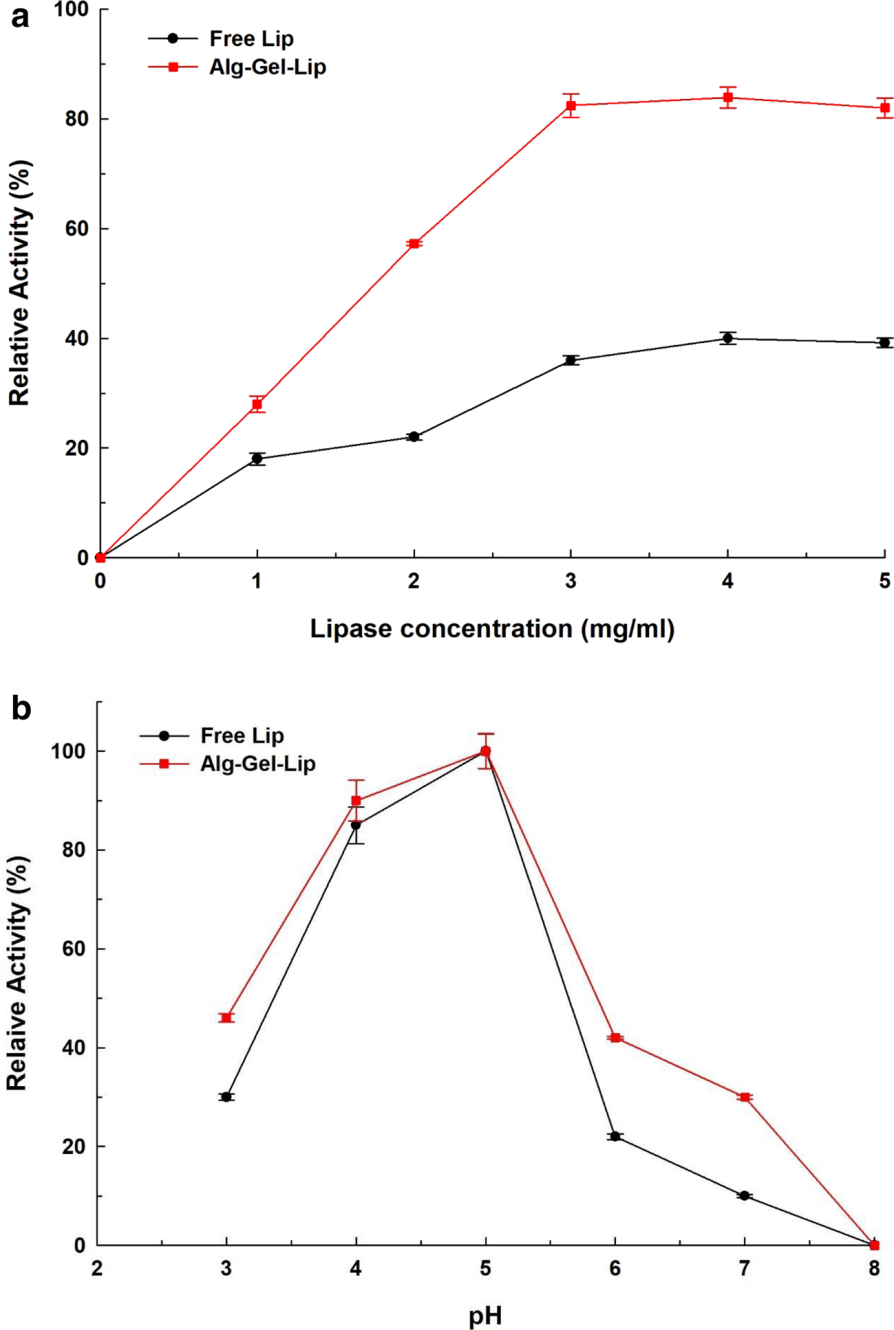
**a** Effect of enzyme concentration on the hydrolysis of olive oil (using 0.0 to 5.0 mg) free Lip or Alg-Gel-Lip solid enzyme/mL reaction mixture with reaction time 3 h). **b** Effect of pH on the catalytic activity of free Lip and immobilized Lip (Alg-Gel-Lip) at different pH values (pH 3.0-pH 9.0); used buffers: 0.05 M Glycine–HCl (3.0–4.0), sodium acetate (4.0–5.0), sodium succinate (6.0–6.5), potassium phosphate (6.5–8.0), Tris–HCl (8.0–9.0,). The lip activity measurement was performed under normal assay conditions. Each point reflects the average of three experimental results ± SD

It is often assumed that the amount of enzyme used in a bioprocess has a substantial impact on its commercial feasibility. Hence, the efficiency and optimal concentration required of the enzyme have a significant impact on the overall cost of the process. Experiments with various enzyme concentrations were carried out to identify the optimum loading enzyme concentration. Using free and immobilized Lip, the effect of enzyme concentration on olive oil hydrolysis was examined, and the percent conversion of olive oil was calculated. (Figure 2a). When utilizing free Lip (3 mg solid enzyme/ml in the reaction mixture) as the biocatalyst, a maximum degree of hydrolysis with 36 percent was achieved. However, increasing the amount of the enzyme to 4 mg solid enzyme/mL resulted in an insignificant (*P* > 0.05) change in the degree of hydrolysis. The data also showed that when Alg-Gel-Lip was used as the biocatalyst, the degree of olive oil hydrolysis increased significantly (*P*< 0.05) from 0.0 to 57.2 percent when 2 mg solid enzyme/mL was used, followed by a significant (*P* < 0.05) increase to 83.9 percent when 4 mg solid enzyme/mL was used. It is expected that as the enzyme concentration increases, the amount of loaded enzyme increases, and at a certain concentration, saturation occurs, limiting further catalysis (Abdel-Mageed et al. 2012, 2018a). Although the use of free Lip resulted in a 40% hydrolysis of olive oil, it required twice as much Lip as Alg-Gel-Lip. In addition, the increase in Lip concentration showed no influence on the degree of hydrolysis of olive oil in either scenario. Furthermore, there was a statistically significant (*P*< 0.05) difference in the degree of olive oil hydrolysis between the free Lip and Alg-Gel-Lip at 2, 3, and 4 mg solid.

Because oleic acid is the most abundant free fatty acid in olive oil, the findings of the Lip activity analysis indicate that the samples with Alg-Gel-Lip, as the biocatalyst, will yield the maximum yield. Complete olive oil hydrolysis was obtained within 60 minutes under ideal experimental circumstances. Similar findings for improved hydrolysis of various vegetable oils following lipases immobilization were also reported in the literature (Table 1). In comparison to previously published experimental work in oil hydrolysis using various immobilized lipases as biocatalysts, these results reveal that a short reaction time is necessary to obtain maximum FFA product. Based on the results obtained, the optimal concentration of free Lip and Alg-Gel-Lip for olive oil hydrolysis was determined to be 4 mg solid enzyme/mL and was used in further experimental studies.

**Table 1:** Comparison of reported experimental hydrolysis studies for free fatty acid production using different immobilized lipases

Feedstock	Microbial Lipase	Immobilization approach	Immobilization efficiency	Reaction temperature (°C)	Reaction time (h)	Hydrolysis (%)	References
Olive oil	*Mucor racemosus*	Adsorption on alginate- hydrogel using sonication	94.5%	37	1	100	Present study
Fish oil	Recombinant *Bacillus subtilis*	Covalent bonding to magnetic nanoparticles	95%	–	–	5	Verma et al.(2019)
Olive oil	*Candida viswanathii*	Adsorption on octyl-agarose support	72.5%	45	–	90	de Almeida et al. (2018)
Fish oil	*Candida* *antarctica* *Rhizomucormiehei*	Hydrophobic chitosan	43% 73.1%	–	30	90 88	Urrutia et al. (2018)
Olive and canola oils	*Candida rugosa*	Covalent bonding to glutaraldehyde-activated polyester fiber	–	35	5	10 (olive oil) 20 (canola oil)	Yiğitoğlu and Temoçin (2010)

#### pH optimum

The pH profile at 3–8 pH range using various buffers for the free and immobilized Lip was examined to provide an understanding of the effect of pH change on the chemical properties of Lip upon immobilization (Figure 2b). Both free Lip and Alg-Gel-Lip showed a maximum activity between pH 4.0 and 5.0 with an optimal activity at pH 5.0. The Lip activity was found to be influenced by pH values greater than 5.0. Alg-Gel-Lip was also found to be more resistant to acid pH values than free Lip. At all measured pH values, Alg-Gel-Lip showed improved hydrolytic activity over the free form. These increased stability results can be attributed to the physicochemical stabilization and protective effect of enzyme immobilization in the hydrogel matrix, where the immobilization process frequently affects the enzyme ionization state, dissociation, and conformational structure. Several authors have previously observed that pH has a significant impact on the stability and activity of immobilized enzymes. (Abdel-Mageed et al. 2019a, b; Arana-Peña et al. 2019). In general, the substitution of carbonate ions or anionic surfactants with hydroxide ions causes the observed loss in activity at alkaline pH values for both free and immobilized Lip. As a result, lipase’s structural arrangement was disrupted and its activity was dramatically reduced at alkaline pH (Aghaei et al. 2020).

#### Temperature optimum

**Fig. 3:**
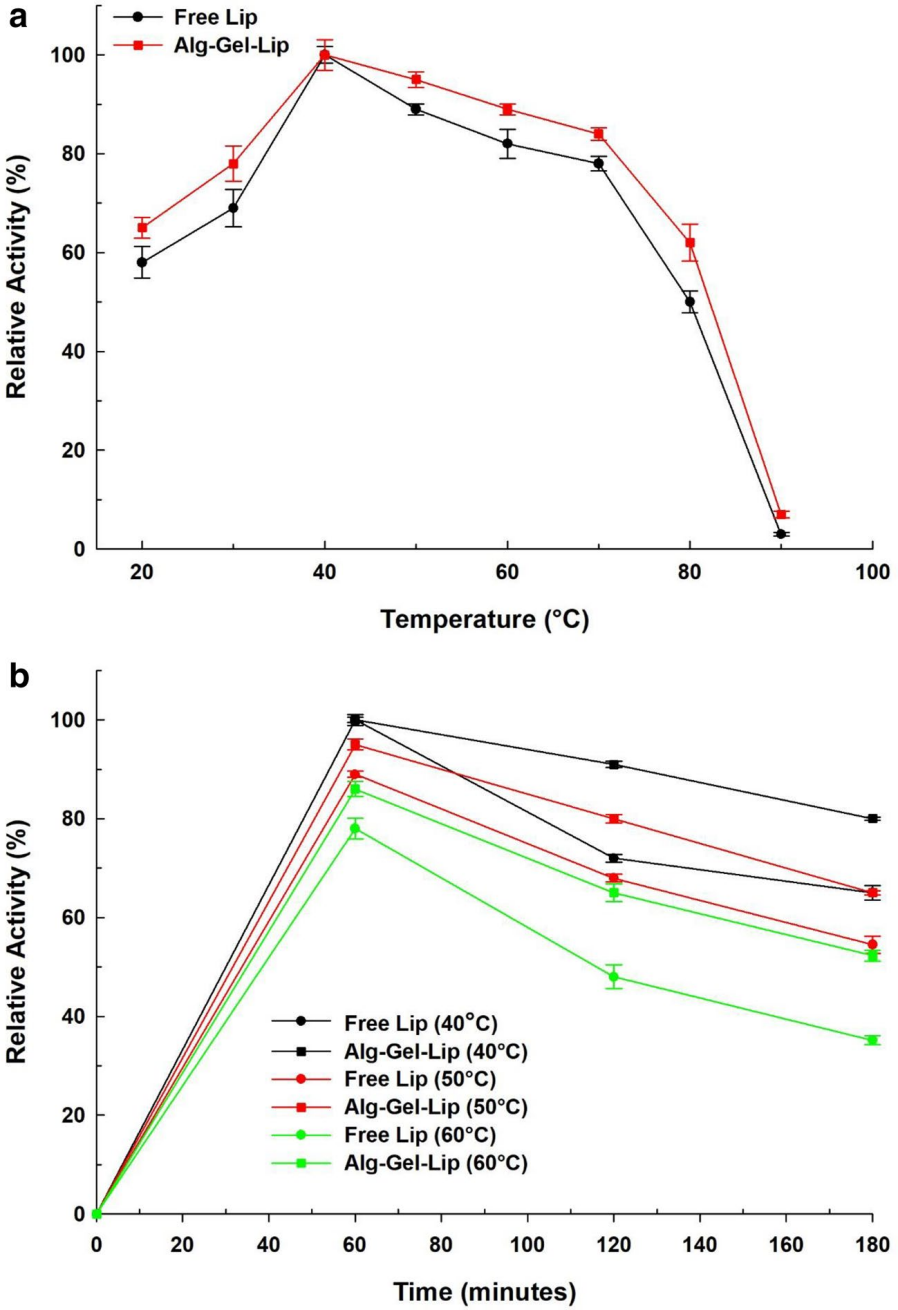
**a** Effect of temperature on the catalytic activity of free Lip and immobilized Lip (Alg-Gel-Lip) at various temperatures from 20-90 °C. **b ** Thermal stability of free Lip and immobilized Lip (Alg-Gel-Lip) after incubation at 40, 50, and 60 °C for 180 min). The lip activity measurement was performed under normal assay conditions. Each point reflects the average of three experimental results ± SD

Temperature is one crucial factor that affects the integrity of the enzyme structure. It is widely documented that maintaining the enzyme's conformational structure is critical for optimizing and maintaining enzyme’s activity (Abdel-Mageed et al. 2012). In this set of experiments, the temperature profile of free and immobilized Lip at temperatures ranging from 20 to 90 °C was carried out to establish the optimum catalytic temperature (Fig. 3a). Both free and immobilized Lip exhibited higher stability at lower temperatures between 20 °C–40 °C and had an optimum temperature at 40 °C. A steeper decline in the activity of the free Lip was observed in contrast to Alg-Gel-Lip at higher temperatures. Alg-Gel-Lip exhibited significant-high activity at high temperatures, where 90% of activity was retained at 60 °C. At all measured temperatures, Alg-Gel-Lip showed improved hydrolytic activity over the free form. The physicochemical stabilization and protective impact upon enzyme immobilization in the hydrogel matrix can be credited to these improved stability outcomes (Abdel-Mageed et al. 2012).

#### Determination of kinetic and thermodynamic parameters

##### Thermal stability

Generally, the distortion of the active site of lipase by heat and pH are the primary reasons for the loss of enzyme catalytic activity. Hence, wide thermal stability is critical for effective industrial enzyme application. It is critical to assess the strengths and limitations of the produced Alg-Gel-Lip biocatalyst because different immobilized enzyme systems exhibit varied thermal stabilities. This study contrasts the differences in thermal stability of free Lip and Alg-Gel-Lip. Using different temperatures (40, 50, and 60 °C), the thermal stability study for both free and immobilized Lip was measured after incubation for 180 min prior to substrate addition. Results showed a higher residual activity reported for the immobilized Lip in comparison to the free Lip at all tested temperatures indicating that the immobilized Lip is more thermally stable (Fig. 3b). A pronounced decline in free Lip activity was detected in contrast to the immobilized Lip in the hydrogel matrix. After 180 min incubation at 40 °C, the residual free Lip activity was 70% whereas the immobilized Lip exhibited 85% residual activity. Furthermore, after 180 minutes of incubation at 60 °C, only 37 % of the free Lip activity was preserved, whereas 59 % of residual activity was found for the immobilized lipase. Hence, it can be concluded that at higher reaction temperatures, coagulation and loss of free lipase activity are more pronounced (Mohd Hussin et al. 2020).

##### Half-life, inactivation constant and activation energy

At a certain temperature, the half-life of an enzyme (t_1/2_) is the time taken for the enzymatic activity to drop to 50% of its initial activity. A longer half-life reflects the enzyme's thermal stability at this temperature, thus improving the probability of successful application in the industry (Abdel-Mageed et al. 2019a, b). The thermal inactivation constant (k_d_) was calculated as previously described in the Methods Section. Whereas, k_d_ is a critical factor for investigating the irreversible effects of enzyme thermal denaturation, a low value of k_d_ or a long half-life (t_1/2_) indicates that the enzyme is thermally stable (Ferreira et al. 2019). The results demonstrate that as temperature increases (40, 50, and 60 °C), the values of t_1/2_ for free Lip (150, 80, and 36 minutes) and Alg-Gel-Lip (650, 260, and 90 minutes) decrease drastically on the one hand, while kd values rise progressively on the other (see Table 2), illustrating the higher irreversible denaturation that comes with temperature rise. The computed K_d_ for free Lip and Alg-Gel-Lip at 40 °C are 0.015 and 0.0082 min^-1^ respectively. These results reveal a greater irreversible denaturation rate of free Lip as temperature increases. These findings can be explained by the fact that the enzyme immobilization support inhibits the enzyme's movement thereby conserving its rigidity and conformational structure as the temperature rises (40 to 60 °C). Hence, immobilized enzyme stability is detected at high temperatures (Aziz et al. 2015).

**Table 2:** Deactivation rate constant (Kd) and half-life (T_1/2_) of free Lip and immobilized Lip (ALg-Gel-Lip) in hydrogel matrix of olive oil hydrolysis

	Parameter	Temperature (K)		
		313	323	333
Free lip	K_d_ (min^−1^)	0.0106	0.0118	0.0154
	T _1/2_ (min)	150	80	36
Alg-gel-lip	K_d_ (min^−1^)	0.0046	0.0069	0.0089
	T _1/2_ min)	650	260	90

Arrhenius Eq. (5), was used to calculate the activation energy (Ed) for the thermal denaturation of Alg-Gel-Lip. E_d_ is the amount of energy required to denature a soluble (native state) enzyme (causing irreversible conformational changes), and a greater value is considered to be a reliable indicator of high enzyme thermal stability. For free Lip, the activation energy (E_d_) calculated using the linear fit to the Arrhenius equation is 22.36 kJ mol^-1^. As a result, the high and positive E_d_ value calculated for Alg-Gel-Lip (87.96 kJ/mol) indicates higher thermostability. Several studies have shown enhanced thermal stability of Lip following immobilization, where the polymeric network protects the enzyme's conformational shape from denaturation (Abdel-Mageed et al. 2019b). These findings also suggest that the biopolymers used in this work have no denaturing effect on Lip—even when subjected to heat stress. Comparable data for improving lipase thermal stability after immobilization can be found in the literature (Ferreira et al. 2019).

##### Kinetic parameters

The performance of free Lip and immobilized Alg-Gel-Lip in terms of the rate and extent of conversion of olive oil into FFA are determined using Michaelis–Menten kinetics. The kinetic data are fitted to the Lineweaver–Burk plots and the kinetic parameters K_m_ and V_max_ and catalytic efficiency and turnover number (K_cat_) are then calculated. Results presented in Table 3 show that the Km value of free Lip (2.2 mM/ml) is slightly lower than that of Alg-Gel-Lip (2.58 mM/ml). Furthermore, the V_max_ value for free Lip is 5.5 mMol oleic acid/ml/min and 5.8 mMol oleic acid/min/ml for Alg-Gel-Lip respectively. Considering both the free and immobilized Lip, the calculated V_max_ values are close to each other. The low K_m_ value with high V_max_ indicates a higher enzyme-substrate affinity and a high reaction rate (Abdel-Mageed et al. 2012). Considering the obtained results, the immobilization process in the hydrogel matrix seems to have a minimum effect on Lip activity in comparison to the free Lip. Immobilization of enzyme generally increases the K_m_ value due to either conformational change in enzyme structure upon immobilization or due to the steric effect and diffusional constraints exerted by the immobilization support that affects the accessibility of substrate to enzyme’s active sites (Abdel-Mageed et al. 2019b, 2021b, a). V_max_ value of an enzyme signifies how fast the enzyme can hydrolyze a completely saturated substrate. Hence, a higher V_max_ value upon immobilization indicates that less substrate is required to be converted to a product per unit of time (Abdel-Mageed et al. 2019b). Other authors have previously found K_m_ and lower V_max_ values after immobilization (Pulat and Akalin 2013; Aghaei et al. 2020). Results demonstrated that the immobilized Lip's V_max_ and turnover number (K_cat_) were larger than the free Lip's which indicate a higher diffusion rate and thus an increase in Lip reaction rate upon immobilization. In contrast to the free enzyme status, it is believed that the hydrogel matrix used to immobilize Lip causes less interfacial interaction with the substrate due to crosslinking. In addition, the catalytic efficiency of immobilized Lip (V_max_/K_m_) is lesser than that of the free enzyme (Table 3). Several studies have similarly reported similar findings following lipase immobilization (Abdel-Mageed et al. 2019a, b).

**Table 3:** Determination of kinetic parameters (K_m_, V_max,_ K_cat_) and enzymatic efficiency of free and immobilized lipase enzyme (Alg-Gel-Lip) for olive oil hydrolysis

Enzyme sample	K_m (_mM/L)	V_max_ (mMol L^−1^ min^−1^)	Enzymatic efficiency (V_max/_ K_m_)	Kcat (S^−1^)
Free lip	2.2	5.5	2.5	3.57
Alg-gel-lip	2.58	5.8	2.24	3.76

The kinetic parameters of the Michaelis–Menten equation for the free and immobilized lipase. Reaction conditions: lipase/support: 4 mg g − 1, pH of immobilized lipase activity = 7, pH of free lipase activity = 7, reaction time: 3 h at 37 °C

#### Reusability and operational stability

**Fig. 4:**
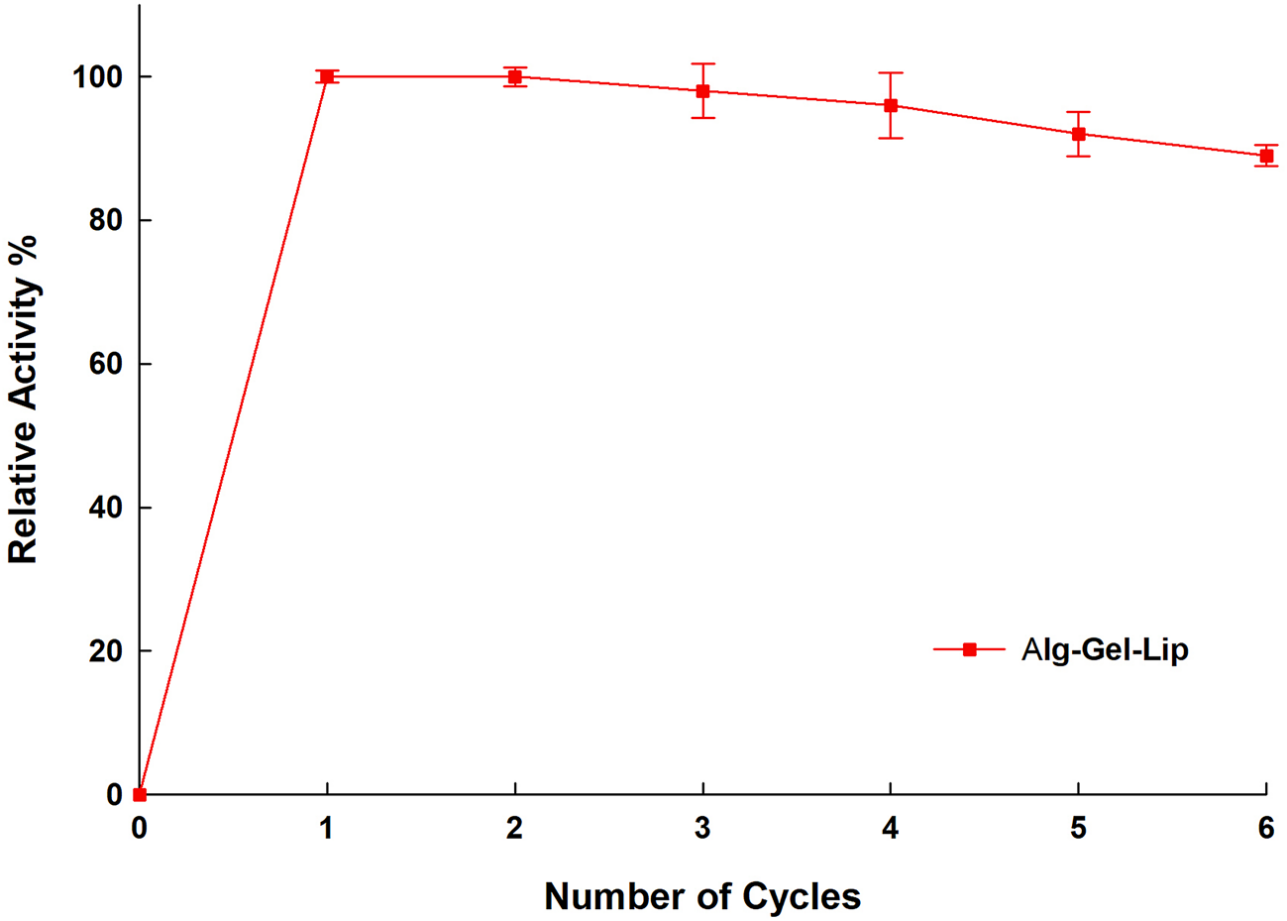
Reusability of immobilized Lip Alg-Gel-Lip (in %) after six repeated hydrolytic cycles (initial activity was considered as 100%). The lip activity measurement was performed under normal assay conditions. Each point reflects the average of three experimental results ± SD

Immobilization approaches are typically employed to surpass the enzymes’ intrinsic liability explicitly under industrial processing. Immobilized enzymes reusability is an important attribute that hinders the process more economically efficient and additionally is not conventional for free enzymes. The reusability of the Alg-Gel-Lip is executed for six consecutive cycles and the results are presented in Fig. 4. After four consecutive hydrolytic cycles, the residual activity for Alg-Gel-Lip was found to be 96%. The high retained activities of Alg-Gel-Lip are affected by hydrophobic interactions between Lip and hydrophobic parts of the support matrix which impedes Lip denaturation. Reusability of Alg-Gel-Lip is therefrore superior to the previous study reported by Huang et al. (2011) which showed that the cellulose membrane immobilized lipase retained only ∼30% residual activity after eight hydrolytic cycles. Similarly, another study reported that immobilized lipase activity decreased to 70% after 15 repeated cycles (Çakmakçi et al. 2017). These results demonstrate that the optimized immobilization process described in this study has successfully created a stabilized-immobilized Alg-Gel-Lip that preserves high enzymatic catalytic efficiency suitable for industrial applications.

#### Storage stability

**Fig. 5:**
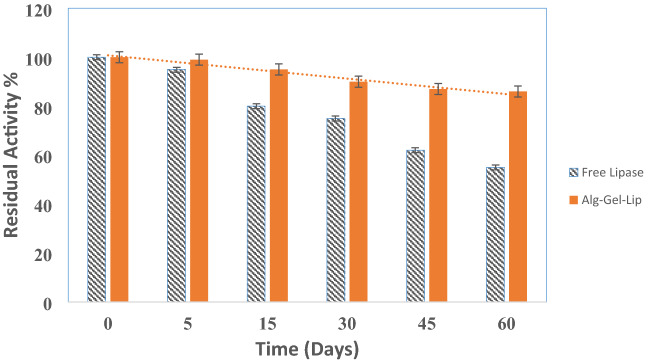
Storage stability of immobilized Lip (Alg-Gel-Lip) upon storage at 5 °C for 60 days (50 mM potassium phosphate buffer, pH 7.0,). Initial activity at day zero was considered as 100%. The lip activity measurement was performed under normal assay conditions. Each point reflects the average of three experimental results ± SD

Storage stability is an important measure of the immobilization process and industrial application performance. Free Lip and immobilized Alg-Gel-Lip were kept at 4 °C in potassium phosphate buffer (0.5 mM, pH 7) for 60 days. An enzyme activity measurement was performed under standard assay conditions at specified time intervals. (Fig. 5) shows that free Lip activity decreases more quickly in comparison to the immobilized form under the same storage conditions. Enzymes are typically stable in their free form for a short period of time. Free Lip retains 80% of its initial activity during the first two weeks and 75% of its activity after 30 days. Immobilized lipase on the hydrogel matrix, on the other hand, demonstrated greater storage durability with 95% residual activity after two weeks and 90 % of the initial activity after 30 days. The improved storage stability of immobilized Lip is bound to the protective effect due to entrapment in the gel matrix, which protects lipase molecules against conformational alterations and preserves its three-dimensional structure during storage (Camilloni et al. 2016). Similar results demonstrating enhanced storage stability of immobilized enzymes are described in the literature (Abdel-Mageed et al. 2018b, 2019a,b).

### Application and Comparative analysis using ultrasonic VS. mechanical stirring method

#### Effect of reaction time

**Fig. 6:**
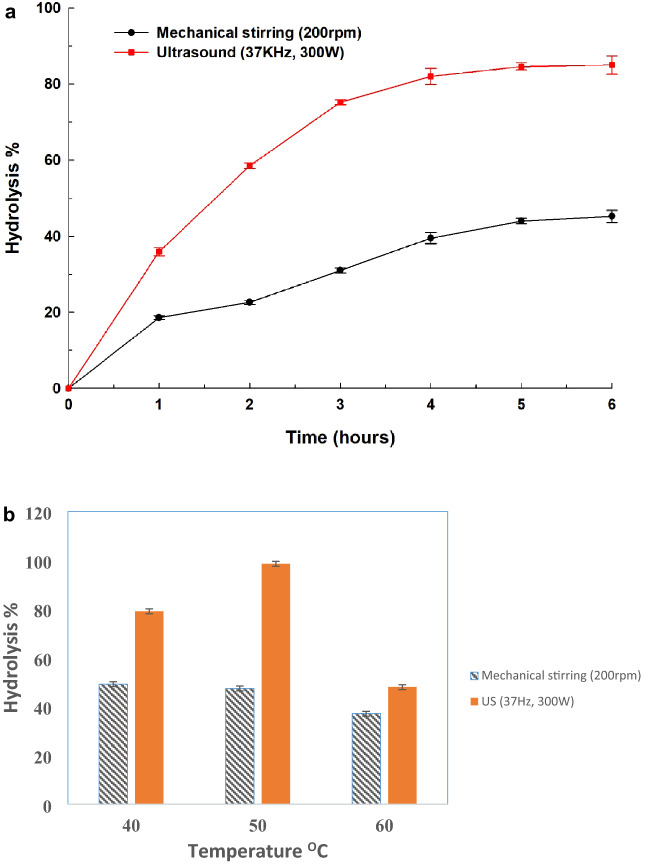
**a** Effect of reaction time on olive oil hydrolysis using Alg-Gel-Lip as the biocatalysts under either mechanical agitation (200 rpm) or ultrasound irradiation (37 Hz, 300 W) using time intervals 0 to 6 h. Initial concentration at time zero was taken as 100%. **b** Effect of varying temperatures on olive oil hydrolysis using Alg-Gel-Lip as the biocatalysts under either mechanical agitation (200 rpm) or ultrasound irradiation (37 Hz, 300 W) using different temperatures from 40 to 60 °C for 3 h. The lip activity measurement was performed under normal assay conditions. Each point reflects the average of three experimental results ± SD

In this study, the prepared biocatalyst is appraised as an attractive green approach for the production of FFA from olive oil hydrolysis. To compare the genuine effect of ultrasound on olive oil hydrolysis using Alg-Gel-Lip biocatalyst, enzyme-catalyzed hydrolytic reactions were carried out under ultrasound and simple conventional mechanical stirring techniques wherein the percentage of the conversion of olive oil was measured. The reaction medium was incubated at 40 °C and was examined in the designated time intervals. The time course for the enzyme-catalyzed hydrolysis of olive oil is presented in (Fig. 6a). The hydrolysis percent of olive oil after 3 and 6 hours was 31 and 45.2 %, respectively, after applying a wide range of reaction times (0 to 6 hours) under mechanical stirring. Ultrasonic irradiation, on the other hand, resulted in a higher reaction yield. Within the first 2 hours of the hydrolysis process, Alg-Gel-Lip Lip catalyzed hydrolysis exhibited a significant increase (*P* < 0.05) in the degree of hydrolysis—from 0.0 to 58.2 %—followed by a significant (*P* < 0.05) increase in the rate of hydrolysis to 85.2 % after another hour. These results could be explained in view of ultrasound cavitation dynamics. When ultrasound energy is applied to a liquid reaction medium, cavitation (due to low-frequency ultrasound) and heating (due to high-frequency ultrasound) are expected to occur (Santos et al. 2009). Cavitation is more favorable for the sequential development, growth, and implosive collapse of small bubbles in liquid media during enzyme-catalyzed bioprocesses. The dynamics of cavity expansion and collapse are influenced by the kind of liquid, dissolved chemicals, liquid temperature, as well as the presence of gasses. It is therefore determined that, in contrast to the collapse of a cavitation bubble in a homogeneous system (liquid-liquid interface), the collapse of a cavitation bubble in heterogeneous systems (example: enzyme system, biomass) is more violent. This is bound to mass transfer constraints at the solid-liquid interface as well as the rapid collapse on/or near the solid surface which creates a forceful liquid jet and/or shock wave that reduces the mass transfer barrier and allows direct contact between the substrate and the enzyme surface. In addition, the rapid breakup of the cavitation bubbles causes significant shear force in the bulk liquid in the immediate region of the substrate which results in a vigorous stirring impact on the immediate liquid layer at the solid-liquid interface where enzyme reactions occur. As a result of the breakdown of the interfacial barrier at the substrate's surface, enhanced mass and heat transfer—of this typically immobile layer—occurs and improves the enzyme's catalytic activity by allowing the enzyme to better reach the substrate's surface (Souza et al. 2020). Simple mechanical stirring, on the other hand, is not an effective method for the immediate solid-liquid interface where the enzymatic reaction occurs (Yachmenev et al. 2009). Moreover, the conformational flexibility of enzymes in solution allows them to precisely align their catalytic active site with the substrate molecules. The correct fit of the substrate to the enzyme's active site has a relatively bigger impact on how well the enzyme catalyze processes.

It is also proposed that the high mechanical stirring impact on the cavitation collapse causes the generated product at the enzyme's surface to be eliminated from the reaction site, thus improving the enzymatic reaction rate (Waghmare and Rathod 2016). Because most of the ultrasound energy is dissipated rather being converted to heat during the cavitation process, it can be concluded that it has no impact on the enzyme macromolecules. Furthermore, lower sonication frequencies generate more cavitation fizzes and hence stronger "jets," which result in more intense stirring of the liquid's border layer at the solid-liquid interface that promotes enzyme catalytic activity.

#### Effect of temperature

The impact of temperature on the degree of hydrolysis is examined at temperatures ranging from 40 to 60 °C using Alg-Gel-Lip as a biocatalyst and ultrasound-assisted olive oil hydrolysis. The results revealled that the degree of hydrolysis of olive oil exhibited a significant increase (*P* < 0.05) from 79.1% at 40 °C to a maximum of 98.6% at 50 °C, then a significant decrease (*P* < 0.05) to 48.1 percent at 60 °C as shown in Fig. 6b. In contrast, Alg-Gel-Lip showed an insignificant (*P* > 0.05) increase in the degree of olive oil hydrolysis from 40 to 50 °C when mechanical agitation is used that was followed by a substantial (< 0.05) decrease to 37.2 percent at 60 °C.

Experimental data, thus, designatesd temperature as a critical parameter for Alg-Gel-Lip, that affects the hydrolysis yield of olive oil upon utilizing ultrasound. In addition, temperature affects the enzyme activity and stability and also the state of the reaction media and/or interface. Hence, while there was a statistically significant (*P* <0.05) difference in the degree of olive oil hydrolysis when utilizing ultrasound at 40 and 50 °C, the degree of hydrolysis when using mechanical stirring at 40 and 50 °C is in contrast insignificant (*P* > 0.05). The increasing rate of olive oil hydrolysis via ultrasound can be related to a decrease in the viscosity of the reaction mass near the enzyme, which can support the formation of the enzyme-substrate complex due to a decrease in mass transfer resistance, as previously demonstrated (Waghmare and Rathod 2016).

Furthermore, the significant reduction in hydrolysis rate at higher temperatures (60 °C) can be linked to disruption of the enzyme's tertiary structure, which resulted in structural denaturation. On the long term, the presence of the denatured enzyme can hinder active enzymes from diffusing at the interface. Moreover, there can be an impact of ultrasound cavitation on reducing hydrolysis with temperature rise since the cavitation effect is reported to be less severe at higher temperatures than it is at lower temperatures (Santos et al. 2009). At high temperatures, in the instance of 60oC, the magnitude of ultrasonic cavitation impact on the reaction mixture was low, which is similar to the surface tension, bubble formation, and mass transfer, wherein as bubble formation is low the mass transfer is relatively lower. Similarly, other authors have reported similar impacts of temperature on ultrasonic-assisted transesterification reactions (Patchimpet et al. 2021).

## Conclusion

The design of fully active immobilized enzymes is a significant and applicable topic of modern research and technology at the present time. This study provided an eco-friendly and economic process for the production of FFA through enzymatic hydrolysis of olive oil catalyzed using crude lipase extract from *Mucor* *racemosus*. Immobilization of lipase ((Alg-Gel-Lip) on biodegradable support (hydrogel matrix) improves the system stability and protects the active site of the enzyme against denaturation. In addition, it enables the separation and the acceleration of the enzyme recovery and reusability. The improved operational, thermal, and storage stability of Alg-Gel-Lip are relevant and therefore significant factors for future industrial applications which signifies the advantages of the examined immobilized lipase system in this study. Although ultrasonic-assisted reactions have been demonstrated as a promising alternative to traditional stirring methods in laboratory settings, scaling up these types of reactions still presents several obstacles. Chemists, chemical engineers, and physicists must work together to build such a big reactor employing ultrasonic equipments with uniform intensity distribution across the entire reactor volume.
